# Production of lovastatin and itaconic acid by *Aspergillus terreus*: a comparative perspective

**DOI:** 10.1007/s11274-017-2206-9

**Published:** 2017-01-19

**Authors:** Tomasz Boruta, Marcin Bizukojc

**Affiliations:** Faculty of Process and Environmental Engineering, Department of Bioprocess Engineering, Lodz University of Technology, ul. Wolczanska 213, 90-924 Lodz, Poland

**Keywords:** *Aspergillus terreus*, Itaconic acid, Lovastatin, Metabolites

## Abstract

*Aspergillus terreus* is a textbook example of an industrially relevant filamentous fungus. It is used for the biotechnological production of two valuable metabolites, namely itaconic acid and lovastatin. Itaconic acid serves as a precursor in polymer industry, whereas lovastatin found its place in the pharmaceutical market as a cholesterol-lowering statin drug and a precursor for semisynthetic statins. Interestingly, their biosynthetic gene clusters were shown to reside in the common genetic neighborhood. Despite the genomic proximity of the underlying biosynthetic genes, the production of lovastatin and itaconic acid was shown to be favored by different factors, especially with respect to pH values of the broth. While there are several reviews on various aspects of lovastatin and itaconic acid production, the survey on growth conditions, biochemistry and morphology related to the formation of these two metabolites has never been presented in the comparative manner. The aim of the current review is to outline the correlations and contrasts with respect to process-related and biochemical discoveries regarding itaconic acid and lovastatin production by *A. terreus*.

## Introduction

The filamentous fungus *Aspergillus terreus* is primarily associated with the biotechnological production of two valuable metabolites, namely itaconic acid and lovastatin. The former has a wide range of applications in polymer manufacturing (Robert and Friebel 2016; Willke and Vorlop 2001), while the latter is used as a cholesterol-lowering drug and a starting material for the production of semisynthetic statins in the pharmaceutical industry (Tobert 2003). These two molecules are the textbook examples of industrially relevant fungal metabolites.

The production of itaconic acid and lovastatin is encoded within the genomic segments referred to as the biosynthetic gene clusters (Brakhage 2013; Keller 2015). The clusters can be described as the groups of neighboring genes collectively responsible for the biosynthesis of a particular metabolite. Following the sequencing of *A. terreus* NIH 2624 genome at the BROAD Institute, the bioinformatic analyses revealed the presence of more than 10,000 putative protein-encoding sequences. Remarkably, it was later observed that the gene clusters corresponding to lovastatin and itaconic acid biosynthesis are situated next to one another in the genome of *A. terreus* (Li et al. 2011). In other words, the two metabolites responsible for the “biotech career” of *A. terreus* were found to be encoded within a relatively small segment of DNA comprised of several genes.

The hierarchical level of genetic organization was discovered to exist in fungal genomes in the form of the so-called superclusters (Wiemann et al. 2013), which can be understood as biosynthetic gene clusters grouped within larger genomic units (“clusters of clusters”). In the light of these findings, it is tempting to speculate that the lovastatin and itaconic acid clusters, which are situated adjacent to one another, may be the members of a coordinately regulated supercluster of great biotechnological importance, shaped and optimized in the course of evolution. However, there is currently no experimental evidence that the two clusters may share a common regulatory mechanism or that their production is jointly coordinated at a molecular level. Despite the adjacent positions of the two biosynthetic gene clusters, the likelihood of the existence of common regulation is rather low. In fact, there is only one literature record regarding the parallel biosynthesis of lovastatin and itaconic acid by an individual strain, namely *A. terreus* ATCC 20542 (Lai et al. 2007). The authors noted that lovastatin production was enhanced when itaconic acid at the concentration of 0.5 g l^− 1^ was supplemented to the medium. It was thus suggested that there might have been a relationship between the biosynthesis of these two molecules. To the best of our knowledge, the biosynthetic co-occurrence of itaconic acid and lovastatin was never reported in subsequent studies. It is likely that the strains isolated for the purpose of lovastatin manufacturing are very poor producers of itaconic acid and vice versa. In the aforementioned study of Lai et al. (2007) the strain *A. terreus* ATCC 20542, a basic lovastatin-producing strain, produced only about 0.5 g l^− 1^ of itaconic acid. This is a very small concentration if compared to the titers exceeding 130 g l^− 1^ obtained with the use of *A. terreus* NRRL 1960 (Karaffa et al. 2015) or *A. terreus* DSM 23081 (Hevekerl et al. 2014b).

In the absence of detailed molecular characterization of the underlying regulatory pathways, the comparative discussion on the production of lovastatin and itaconic acid can be attempted on the basis of bioprocess-related observations originating from industrial and academic optimization studies. Thus, the aim of this mini-review is to outline the similarities and differences with respect to the conditions favoring the production of lovastatin and itaconic acid by *A. terreus*. For the purpose of comparison, only the selected aspects of biosynthesis are discussed here, however the readers may consult the previous reviews on the production of lovastatin (Barrios-Gonzalez and Miranda 2010; Bizukojc and Ledakowicz 2015; Manzoni and Rollini 2002; Mulder et al. 2015; Subhan et al. 2016) and itaconic acid (Klement and Buchs 2013; Okabe et al. 2009; Steiger et al. 2013; Willke and Vorlop 2001) for further insights. While the influence of the respective parameters on the productivity of the process is always strain-specific and depends on the applied cultivation strategy, certain generalizations can still be made to open the door for the comparative discussion on lovastatin and itaconic acid production. The formulation of such general remarks is attempted in the current review.

## Biosynthesis

The molecules of lovastatin and itaconic acid are markedly different with respect to chemical structure and originate from secondary and primary metabolism, respectively. The biochemistry of itaconic acid production was the subject of the previous review (Steiger et al. 2013). Itaconic acid is an unsaturated dicarboxylic acid, which is formed via decarboxylation of *cis*-aconitic acid in the reaction catalyzed by *cis*-aconitate decarboxylase (CadA) (Bonnarme et al. 1995; Jaklitsch et al. 1991; Kanamasa et al. 2008; Li et al. 2011; Winskill 1983). Other proteins important for the biosynthesis of itaconic acid are the mitochondrial *cis*-aconitic acid transporter (MttA) and a putative major facilitator superfamily transporter. Importantly, a pathway involves the MttA-mediated transport of *cis*-aconitic acid from the mitochondrion to the cytosol, where the decarboxylation leading to itaconic acid occurs (Steiger et al. 2016). Li et al. (2011, 2013) employed a transcriptomic approach to identify the genes associated with itaconic acid production. In addition to the itaconic acid gene cluster itself, the authors reported a number of genes involved in glycolysis, pentose phosphate pathway, production of vitamins and copper transport.

The biosynthetic route leading to lovastatin is much more complex. It is based on the action of two multi-domain polyketide synthases (PKS), namely the lovastatin nonaketide synthase (LovB) and the lovastatin diketide synthase (LovF), responsible for assembling the carbon skeleton of lovastatin using the acetyl-CoA and malonyl-CoA units. Additionally, a number of other enzymes participate in the so-called *post-PKS tailoring* steps leading to the final structure of lovastatin. The pathway proceeds through a number of intermediates, including 4a,5-dihydromonacolin L, 3α-hydroxy-3,5-dihydromonacolin L, monacolin L and monacolin J (Alberts et al. 1980; Barriuso et al. 2011; Cacho et al. 2015; Kennedy et al. 1999; Xu et al. 2013).

**Fig. 1:**
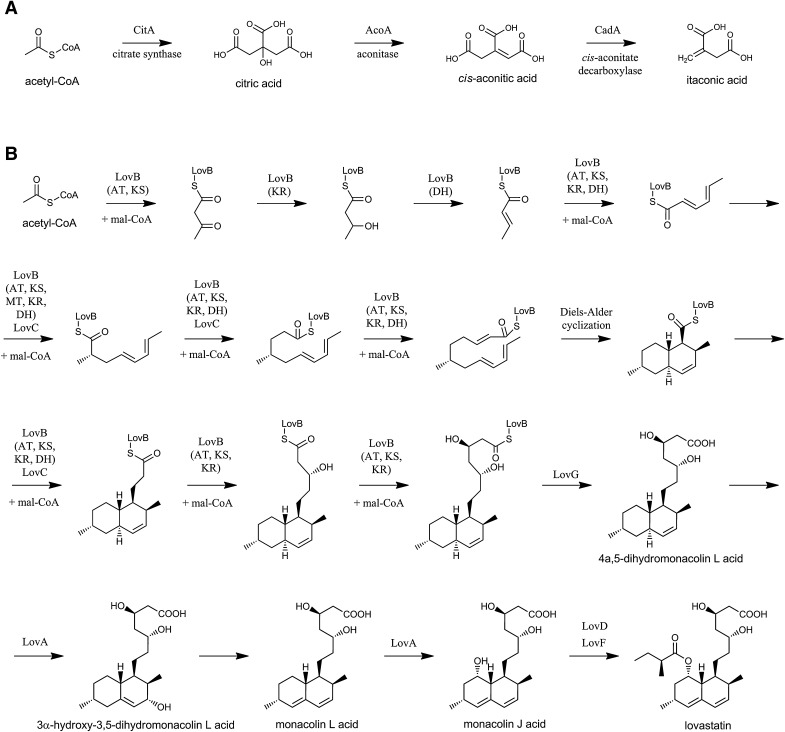
Pathways of itaconic acid (**a**) and lovastatin (**b**) biosynthesis in *Aspergillus terreus* (Ames et al. 2012; Bentley and Thiessen 1957; Kennedy et al. 1999; Steiger et al. 2013). The enzymes participating in the respective steps are indicated. The catalytic domains of lovastatin nonaketide synthase LovB are shown in brackets. *ACP* acyl carrier protein; *AT* acyltransferase; *CadA cis*-aconitic acid decarboxylase; *DH* dehydratase; *KS* β-ketoacyl synthase; *KR* ketoreductase; *LovA* cytochrome P450 monooxygenase; *LovB* lovastatin nonaketide synthase; *LovC* enoyl reductase; *LovD* acyl transferase; *LovF* lovastatin diketide synthase; *mal-CoA* malonyl-CoA; *MT* methyltransferase

The difference in complexity between the metabolic pathways leading to lovastatin and itaconic acid is illustrated in Fig. 1. For comparison, the molecule of acetyl-CoA is depicted as the starting point of both pathways in order to demonstrate the relationship of the presented biosynthetic routes to the respective core metabolic precursors.

Acetyl-CoA is one of the key precursors situated at the intersection of many metabolic pathways. It fuels the carbon building blocks for the biosynthesis of fatty acids and the intermediates of the citric acid cycle. It is also involved in the regulation of metabolic activity within the cell. Importantly, it provides a link between primary and secondary metabolism by delivering the carbon skeleton for diverse groups of secondary metabolites, e.g. polyketides and terpenes (Chiang et al. 2010; Shi and Tu 2015). However, the regulation of its distribution between primary and secondary pathways remains to be elucidated, primarily due to the complexity of the regulatory machinery involved in secondary metabolism, which is far from being fully understood (Brakhage 2013).

As already mentioned in the introduction, due to the adjacency of itaconic acid and lovastatin gene clusters, one may speculate about the possibility of common regulation of biosynthesis of these two metabolites. Importantly, itaconic acid and lovastatin are related to different branches of metabolic machinery, namely to primary and secondary metabolism, respectively. In contrast, the fungal biosynthetic gene supercluster described in literature (Wiemann et al. 2013) encompasses the genes associated with the formation of secondary metabolites. To the best of our knowledge, the fungal supercluster involving the genes of primary and secondary metabolism has never been reported and, accordingly, the common regulation of itaconic acid and lovastatin production appears rather unlikely despite the adjacency of respective genomic segments.

The maximal reported titers of itaconic acid are at the level of 140 g l^− 1^. Accordingly, itaconic acid is considered a commodity chemical. In contrast, the typically reached lovastatin concentration values are below 1 g l^− 1^ and the metabolite itself represents the group of high-value fine chemicals. The titers of lovastatin and itaconic acid are of different magnitudes. This fact makes the common regulation of their biosynthesis even less likely.

The biosynthesis of lovastatin is controlled within the regulatory framework of secondary metabolism (Mulder et al. 2015). Regulation of fungal secondary metabolism involves a number of global and cluster-specific regulators, e.g. a global regulator LaeA and Zn(II)_2_Cys_6_ transcription factors, participating in the complex regulatory and signaling pathways and responding to diverse environmental stimuli (Knox and Keller 2015). Bok and Keller (2004) proved that LaeA is a global regulator involved in the expression of lovastatin biosynthetic genes. Furthermore, one of the proteins encoded in the lovastatin biosynthetic gene cluster, namely LovE, has a zinc finger domain typically found in Zn(II)_2_Cys_6_ regulators (Kennedy et al. 1999). Importantly, it was shown that the onset of lovastatin production coincides with the increase of reactive oxygen species (ROS) and down-regulation of *sod1* (a gene encoding the oxidative stress defense enzyme) observed during the idiophase (Miranda et al. 2013). This study also demonstrated the importance of mass transfer of oxygen in the cultivation broth and within the mycelia to promote the oxidative state associated with lovastatin biosynthesis. It was then suggested that a transcription factor Yap1 could provide a link between the initiation of lovastatin production and the accumulation of ROS. Notably, the *yap1* gene was highly expressed during trophophase but down-regulated during idiophase (Miranda et al. 2014).

In order to activate the PKS enzyme, the post-translational addition of phosphopantetheinyl group to the acyl carrier protein (ACP) domain of the PKS by 4′phosphopantetheinyl transferase (PPTase) is required (Chiang et al. 2010). Interestingly, Márquez-Fernández et al. (2007) reported that that a single PPTase is likely to be involved in the activation of all polyketide synthases in *Aspergillus nidulans*. Since the formation of lovastatin is dependent on the catalytic activity of two polyketide synthases (Hendrickson et al. 1999), their control is crucial for triggering the underlying biosynthetic pathway.

It was suggested previously that the acidification of surroundings with itaconic acid may be an important survival strategy against competitors (Magnuson and Lasure 2004). Lovastatin is an inhibitor of the key step of the cholesterol biosynthesis pathway, namely the reaction catalyzed by (S)-3-hydroxy-3-methylglutaryl-CoA reductase (Endo 2010) and exhibits antifungal and antiparasitic activity (Keller 2015). So, both itaconic acid and lovastatin can be regarded as chemical means of gaining evolutionary advantage in the environmental niche, albeit the mechanisms of providing the advantage and the scale of biosynthesis are markedly different for the two molecules. Supposedly, the relatively low levels of lovastatin secreted by *A. terreus* proved to sufficient to inhibit the proliferation of competing microorganisms in the natural habitat and the evolutionary conservation of the underlying biosynthetic pathway was justified in this species.

## Initial pH value of growth medium

The initial pH values published in relation with itaconic acid production vary significantly in the range from 1.6 (Lockwood and Moyer 1949) to 5.9 (Gyamerah 1995b). The experiments of Rychtera and Wase (1981) involving the strain *A. terreus* NRRL 1960 led to a conclusion that the initial pH value optimal for itaconic acid production is equal to 3.1. Following these findings, the initial pH of 3.1 was later applied in numerous itaconic acid-related experiments (Gao et al. 2014; Hevekerl et al. 2014a; Kautola et al. 1991; Kuenz et al. 2012). The subject was further elaborated by Hevekerl et al. (2014b), who tested 6 different levels of initial pH, ranging from 1.9 to 4.9, in the itaconic acid-oriented cultivation of *A. terreus* DSM 23081. Due to the fact that the manipulation of the initial pH value did not lead to any improvements in performance and seemed to be of secondary importance, the authors decided to conduct their further experiments according to the previous recommendations of Rychtera and Wase (1981) at the initial pH of 3.1. It needs to be emphasized that in all cases examined by Hevekerl et al. (2014b) the onset of product biosynthesis corresponded to the time when the pH decreased to the level of 2.1. The decrease of pH value to about 2 is a typical behavior observed at the start of itaconic acid production (Willke and Vorlop 2001).

Despite the generally accepted approach of starting the lovastatin production at pH 6.5 (reviewed by Mulder et al. 2015), it was demonstrated by Osman et al. (2011) and Bizukojc et al. (2012) that applying even higher initial pH values of 7.5 and 8.5, respectively, can lead to the enhanced biosynthesis of lovastatin. On the other hand, lowering the initial pH value to 4.5 or 3.5 resulted in the strongly inhibited biosynthesis of lovastatin by *A. terreus* ATCC 20542 (Bizukojc et al. 2012). Apparently, the preferred initial pH value for lovastatin production is situated higher on the pH scale than for the formation of itaconic acid. Shifting the pH value in the course of the cultivation should, in principle, allow for the production of both metabolites in the sequential manner. For example, an initial low pH itaconic acid production phase can be followed by a higher pH lovastatin production phase. Even though pH shifts have been tested during itaconic acid production, the presence of lovastatin in the broth have not been assayed (Hevekerl et al. 2014b). The mixed cultivation approach involving pH shifts could provide valuable insights into the itaconic acid and lovastatin biosynthesis occurring in a single strain, provided the concentration values of both metabolites are monitored throughout the cultivation process. Still, such approach has not been explored.

## Control of pH value

Keeping the concentration of hydrogen ions within a certain range or at a fixed value during the cultivation is regarded by some authors to be an effective approach to increase titer, productivity and yield of itaconic acid. Several optimization strategies involving the control of acidity in *A. terreus* cultures were proposed. As an alternative to continuous pH control, the individual adjustment of pH in the chosen time point of the run can be performed to influence the outcome of the process.

The examples of early descriptions and suggestions regarding pH control can be found in literature dating back to 1940s. Lockwood and Moyer (1949) recommended to maintain pH value within the range between 1.4 and 2.4 in order to avoid itaconic acid decomposition during the cultivation. A slightly different method was applied by Nelson et al. (1952), who kept pH value within the narrower range between 1.8 and 2.0. Later, Nubel and Ratajak (1962) described an approach involving partial neutralization of the broth with lime up to pH 3.8, which was conducted when the content of itaconic acid reached the level from 20 to 50 g l^− 1^. The final product was described as substantially free of other organic acids (Nubel and Ratajak 1962). To eliminate the formation of by-products, namely succinic acid and itatartaric acid, Batti (1964) recommended to keep pH value between 3 and 5 after the itaconic acid concentration in the broth reached about 50 g l^− 1^.

In the aforementioned studies (Lockwood and Moyer 1949; Nelson et al. 1952; Nubel and Ratajak 1962; Batti 1964) the importance of controlling pH levels during itaconic acid production was clearly highlighted, however the formulated recommendations differed significantly among the authors. The need for detailed experimental studies directly addressing the aspects of pH control was evident. Riscaldati et al. (2000) tested a number of diverse cultivation conditions involving pH control and, in the light of the gathered data, suggested to keep pH value at 2.8 in order to maximize itaconic acid production by *A. terreus* NRRL 1960. Importantly, the authors also demonstrated the significance of stirring speed in this context, as the final product concentration at the controlled pH of 2.8 varied from 17.7 g l^− 1^ at 320 rpm to 43.1 g l^− 1^ at 400 rpm. The highest concentration of itaconic acid obtained in the study (57.2 g l^− 1^) was recorded at the controlled pH value of 2.4 and stirring speed of 320 rpm, but due to a relatively low productivity these conditions were not regarded as optimal (Riscaldati et al. 2000). In a different study, Rychtera and Wase (1981) noted the decrease in itaconic acid production by *A. terreus* NRRL 1960 when pH value was above 3.1.

Despite the existence of several reports that indicated the advantages of pH control during itaconic acid production, there are examples of successful cultivations conducted without any pH modifications that led to relatively high product titers ranging from about 90 to 130 g l^− 1^ (Kuenz et al. 2012; Hevekerl et al. 2014a; Karaffa et al. 2015). What is more, one may encounter examples of published data sets supporting the idea that pH control can negatively affect the process. For instance, in a study of Li et al. (2011) the cultivation of *A. terreus* NRRL 1960 without pH control resulted in the final itaconic acid concentration about 3.5 times higher than that in the corresponding process performed with the same initial pH but with its continuous control at 3.5. Recently, Hevekerl et al. (2014b) observed that pH control at the level equal to 3 maintained from the onset of cultivation resulted in a low final product concentration of 17 g l^− 1^ and clearly had a negative impact on the biosynthesis of itaconic acid by *A. terreus* DSM 23081. In the same study, when pH control at the level equal to 3 was initiated not at the beginning of cultivation but after the onset of product formation, the highest reported itaconic acid titer of 146 g l^− 1^ was reached. As pointed out by Hevekerl et al. (2014b), it had been previously suggested that the initial growth period at lower pH is required for the cells to develop the biochemical machinery responsible for itaconic acid biosynthesis (Larsen and Eimhjellen 1955).

While certain studies indicated that a poorly designed pH control strategy may have a negative impact on the production of itaconic acid (Hevekerl et al. 2014b; Li et al. 2011), it was also demonstrated that a well-chosen pH control scheme is a valuable approach of process optimization, provided that certain factors are taken into consideration. These factors include the careful choice of the pH value itself and the moment of pH control initiation (Hevekerl et al. 2014b). Furthermore, other process parameters, e.g. stirring speed, should be monitored in concert with pH in order to reach satisfactory product concentration (Riscaldati et al. 2000). Whereas previous experiments indicated that maintaining a constant value of pH is not a prerequisite for attaining high itaconic acid titers and its importance is rather debatable in this context, the adjustment of pH was shown to reduce the formation of by-products (Batti 1964) and increase the solubility of itaconic acid in the broth (Hevekerl et al. 2014b).

Control of pH during lovastatin production has been applied in several studies. Whenever the authors decided to maintain the control, the pH value was generally within the range of pH 5.8–7.8 (Bizukojc and Ledakowicz 2008; Kumar et al. 2000; Lai et al. 2005; Novak et al. 1997; Pawlak et al. 2012; Pawlak and Bizukojc 2013). Clearly, this interval does not overlap with the more acidic conditions close to pH value between 2 and 3, typically employed for establishing high-yield itaconic acid production processes.

Interestingly, the pH regime associated with maximal itaconic acid production do not correspond with the pH value optimal with respect to *A. terreus* biomass yield, which was determined by Mathan et al. (2013) to be around pH 5.5. It indicates that itaconic acid production is triggered at the conditions suboptimal for growth. It is possible that the formation of itaconic acid serves as a metabolic strategy to utilize the surplus of citric acid intermediates in growth-limiting conditions.

Similarly as in the case of itaconic acid production, the significance of pH control with regard to the biosynthesis of lovastatin is rather controversial. Lai et al. (2005) addressed this subject in a series of experiments involving *A. terreus* ATCC 20542. The control of pH value in the range between 5.5 and 7.5 was applied starting from the 48 h of the cultivation. In comparison with the run performed without the pH control, no improvements in terms of lovastatin titers were recorded at pH level equal to 6.5, whereas at pH levels equal to 5.5 and 7.5 the pH control had a visibly negative impact on the process. While the study of Lai et al. (2005) did not provide any justification for controlling the level of pH during lovastatin production, this approach was then advocated by Bizukojc and Ledakowicz (2008), who described the positive impact of pH control on lovastatin production by *A. terreus* ATCC 20542. Specifically, keeping the pH at the level of 7.6 or 7.8 starting from the 24 h by using sodium and potassium carbonate allowed for the suppression of the production of (+)-geodin, a secondary metabolite often found to co-occur with lovastatin in the broth (Askenazi et al. 2003; Bizukojc and Ledakowicz 2007). In the light of these findings, the reduction of by-product formation can be viewed as the main rationale for applying pH control during lovastatin production.

Considering the aforementioned studies it is tempting to generalize that, in the words of Dowdells et al. (2010), “*Aspergillus terreus* produces itaconic acid at low pH but lovastatin (…) at higher pH”. Indeed, the experimental results obtained so far strongly indicate that the optimal values of pH for itaconic acid and lovastatin differ significantly. However, as discussed in the current review, the pH value is merely one of the factors affecting the biosynthesis of these two molecules. Other cultivation parameters and, most importantly, the associated regulatory mechanisms are also of great importance in this context and should not be overlooked.

The production of itaconic acid is not the only fungal cultivation that requires low pH values. The best-studied example of a process proceeding at low pH values is the production of citric acid by *Aspergillus niger*. Both citric acid and the precursor of itaconic acid, namely *cis*-aconitic acid, originate from the citric acid cycle. As reviewed by Klement and Buchs (2013), itaconic acid production is sometimes regarded as “citric acid production, but in the presence of a *cis*-aconitate decarboxylase”. During the production of citric acid the pH value of the broth must be kept below pH 2.5 to achieve product accumulation (Kubicek and Karaffa 2006). At low pH values the formation of other organic acids, namely gluconic acid and oxalic acid, is suppressed and the risk of contamination is highly reduced (Karaffa and Kubicek 2003; Papagianni 2007). Similarly, the significance of low pH with respect to preventing by-product formation has also been described for the production of itaconic acid (Batti 1964). Due to the p*K*_a_ values of citric acid, equal to 3.1, 4.7 and 6.4, the accumulation of the certain amount of the product in the medium leads to the automatic decrease of pH value to 1.8, unless the medium is strongly buffered, e.g. in the presence of glutamate (Kubicek and Karaffa 2006). The p*K*_a_ values of itaconic acid (3.8 and 5.5) are within the range of p*K*_a_ values exhibited by citric acid (Mondala 2015) and can be associated with the drop of the pH value at the onset of itaconic acid accumulation (Hevekerl et al. 2014b).

The dedicated enzyme of the itaconic acid biosynthesis pathway, *cis*-aconitate decarboxylase (CadA), was demonstrated to exhibit maximal activity at pH 6.2. The activity declined significantly up to pH 7.5. Below pH 4.5 the enzyme was inactive (Dwiarti et al. 2002). However, it should be noted that the enzyme is localized in the cytosol (Jaklitsch et al. 1991) and is not directly exposed to low extracellular pH values. In contrast, glucose oxidase, participating in the formation of gluconic acid as a by-product of citric acid production, is susceptible to external pH values due to its extracellular localization. Hence, the formation of gluconic acid is inhibited by maintaining low pH value of the cultivation medium during citric acid production (Kubicek and Karaffa 2006).

Lockwood and Reeves (1945) noted the inhibitory effect of itaconic acid on its biosynthesis. Kanamasa et al. (2008) proved that the transcription of *cadA* is not affected by itaconic acid, so there is no feedback inhibition at the transcription level of *cadA*. However, *cis*-aconitate decarboxylase was shown to be inhibited by Zn^2+^, Hg^+^, Cu^2+^, *p*-chloromercuribenzoate and 5,5′-dithio-bis(2-nitrobenzoate) (Dwiarti et al. 2002). As noted by Klement and Buchs (2013), the regulation of *cis*-aconitate decarboxylase is still not understood and remains to be elucidated.

Interestingly, the requirement for low extracellular pH as a prerequisite for itaconic acid production is not conserved across the fungal kingdom. For instance, according to Klement et al. (2012), the fungus *Ustilago maydis* performs biosynthesis of itaconic acid within the pH range of 4.5–6.0. In addition, unlike *A. terreus*, it uses an alternative biosynthetic pathway involving *trans*-aconitate as an intermediate (Geiser et al. 2016).

The association of the biosynthesis of itaconic acid and lovastatin with different growth conditions must have provided a certain sort of evolutionary advantage for the producing organism itself. One may speculate that the niche-dependent different biosynthesis of these metabolites supported the proliferation of *A. terreus* strains in diverse habitats. Lowering the pH value of the surroundings with itaconic acid and inhibiting the growth of competing microbes with lovastatin can be both viewed as the established survival strategies employed under different environmental conditions and encoded in the genome of *A. terreus* in the course of evolution.

## Agitation and aeration

The cellular machinery responsible for the biosynthesis of itaconic acid and lovastatin, as well as the growth of *A. terreus* itself, are heavily dependent on oxygen supply. Accordingly, applying well-designed agitation and aeration strategies is a key to achieve industrially relevant productivities and titers. Since the formation of fungal metabolites is significantly influenced by morphology, it is crucial to determine an optimal agitation speed and aeration rate that provide a balance between delivering sufficient amounts of oxygen to the cells and avoiding high levels of mechanical stress.

The studies on itaconic acid and lovastatin production clearly demonstrate that, in general, insufficient agitation leads to hindered product formation due to the shortages in oxygen supply. On the contrary, if the stirring speeds are too high, the negative impact associated with shear stress dominates over the potential advantages of high dissolved oxygen tensions and, as a consequence, the final product titers are rather low. The examples of relevant studies addressing the issue of optimal agitation during itaconic acid and lovastatin production are provided below.

Park et al. (1993) applied stirring speeds ranging from 200 to 400 rpm in order to evaluate the influence of agitation on itaconic acid production by *A. terreus* IFO6365. Throughout the experiment, the aeration rate, expressed in the units of *vvm* (ratio of air flow rate and the volume of the bioreactor), was fixed at 0.5 l_air_ l^− 1^ min^− 1^. It was observed that the stirring speed of 200 rpm was too low to provide sufficient oxygen for the efficient itaconic acid production and, as a consequence, the final titer was below 20 g l^− 1^. It was reflected by the fact that at the agitation speed of 200 rpm the dissolved oxygen concentration decreased to zero after 6 h of cultivation. At the stirring speed of 400 rpm the final concentration of the target metabolite reached 32.3 g l^− 1^ and the microscopic examination revealed the damage of mycelia due to the mechanical stress. The highest titer of itaconic acid (49.4 g l^− 1^) was achieved at the moderate stirring speed of 300 rpm. Under these conditions, the compromise between providing sufficient agitation and avoiding excessive shear stress was achieved. In addition, the authors evaluated the effect of dissolved oxygen (DO) concentration on the production of itaconic acid. In these experiments stirring speed was fixed at 300 rpm and aeration rate was manipulated to reach the anticipated level of DO, namely 20, 40 and 60%. The final concentration of the product was equal to 48.5, 45.2 and 52 g l^− 1^, respectively. Since the achieved product titers were similar for all three examined levels of DO, it was clear that increasing the aeration rate above a certain level was unnecessary in the context of itaconic acid formation (Park et al. 1993). In a different study, changing the stirring speed from 320 to 400 rpm at pH 2.8 allowed for the elevation of the final product titer in the cultures of *A. terreus* NRRL 1960 from 17.7 to 43.1 g l^− 1^, respectively (Riscaldati et al. 2000). However, further increase of the stirring speed to 480 rpm was a step backwards in terms of itaconic acid concentration, which was found to be equal to 33.1 g l^− 1^. The presented trade-off between sufficient agitation and maintaining adequate morphological forms was analogous to the one demonstrated by Park et al. (1993).

Looking beyond the morphological aspects, Pfeifer et al. (1952) pointed out that operating at high agitation and high aeration rates may be problematic due to excessive foaming of the culture broth. Therefore, the agitation and aeration during itaconic acid production were recommended to be increased only up to a certain point. The authors used the strain *A. terreus* NRRL 1960 and conducted their experiments at a pilot plant scale of 200 and 400 gallons (Pfeifer et al. 1952).

The recommendation of conducting the cultivation at the “sufficient, but not too high” agitation speed and aeration rate, formulated towards itaconic acid production, applies equally well to lovastatin biosynthesis. Lai et al. (2005) monitored lovastatin formation by *A. terreus* ATCC 20542 at 225, 325 and 425 rpm. Not surprisingly, the highest titer of 0.305 g l^− 1^ was observed at the moderate stirring speed of 325 rpm. The authors performed the second series of experiments to examine the influence of dissolved oxygen control on lovastatin biosynthesis. The cultivation was conducted at the DO controlled at 10, 20, 30 and 40%. It turned out that controlling the DO at 20% proved to be an optimal choice in this case, leading to the titer of 0.458 g l^− 1^. In the runs with the DO maintained at 10% the oxygen supply turned out to be insufficient, whereas at 30 and 40% the titers were low due to the impact of mechanical stress (Lai et al. 2005). A comparable phenomenon was observed in the cultures of *A. terreus* 20541 by Novak et al. (1997). This time, however, the best-yielding process was conducted at the controlled DO of 70%, while the DO values of 35 and 80% were found to be, respectively, too low and too high to allow for the efficient lovastatin production. Despite the discrepancies in the suggested optimal DO values, the results of Lai et al. (2005) and Novak et al. (1997) supported the same common principle that the level of DO control needs to be carefully adjusted to prevent the damage of mycelia. The morphological issues of lovastatin and itaconic acid production are further discussed in the next section.

In a different study, Bizukojc and Ledakowicz (2008) observed that the increase of aeration rate negatively affects lovastatin production by *A. terreus* ATCC 20542 by promoting the formation of (+)-geodin. This behavior was later confirmed by Boruta and Bizukojc (2016), whose results indicated that the intensive aeration scheme induced the production of (+)-geodin and terrein at the cost of lovastatin, which was found to be present at the noticeably lower concentration compared with the less aerated cultures.

## Fungal morphology

Morphology is one of the key process-related parameters of submerged fungal cultivations (Grimm et al. 2005; Kossen 2000; Wucherpfennig et al. 2010). Generally, the fungus cultivated in the agitated liquid medium may proliferate in the form of dispersed hyphae, clumps or pellets (Gao et al. 2014). The morphological form of a cultivated microorganism depends on a wide range of factors, including medium composition, temperature, pH, oxygen supply and, importantly, mechanical stress exerted by agitation. There are no general rules regarding the mycelial forms recommended for the efficient biosynthesis of metabolites or enzymes. While for certain products the formation of pellets is a prerequisite for achieving high titers and productivities, the biosynthesis of some other molecules may be favored by the presence of dispersed hyphae (Papagianni 2004).

The detailed studies addressing the influence of morphological forms on the production of itaconic acid were performed by Gyamerah (1995a) and Gao et al. (2014). According to Gyamerah (1995a), itaconic acid formation by *A. terreus* NRRL 1960 was favored in the presence of small and frayed pellets with the diameter in the range 0.1–0.5 mm. In the second study involving the strain *A. terreus* FMME033, Gao et al. (2014) noted that clumps with the diameter of 0.4–0.5 mm were preferred over pellets. Therefore, the conclusions of Gao et al. (2014) were in agreement with the ones presented by Gyamerah (1995a) with respect to the size, but not the type, of the morphological form. A markedly different opinion was presented by Rychtera and Wase (1981), who suggested to avoid the formation of pellets during itaconic acid production.

In the case of lovastatin biosynthesis, the search for optimal morphology have been undertaken by several research groups (Bizukojc and Ledakowicz 2010; Casas Lopez et al. 2005; Gbewonyo et al. 1992; Gupta et al. 2007; Jia et al. 2009; Rodriguez Porcel et al. 2006a, b). Although it is generally agreed that pellets are the morphological form desired for lovastatin formation, there is a great variety with respect to the suggested optimal diameters of the pellets, which were proposed, for example, to be less than 1.5 mm (Bizukojc and Ledakowicz 2010), 0.95 mm on average (Lai et al. 2005) or within the range 1.8–2.0 mm (Gupta et al. 2007).

Due to the discrepancies regarding the recommended optimal morphologies, it is difficult to make comparisons between lovastatin and itaconic acid production in this particular aspect. Nevertheless, the biosynthesis of both metabolites can be induced by using similar methods of morphology engineering. The traditional strategies of controlling the morphological forms, involving the adjustment of mechanical stress and the number of spores, have been attempted in both cases (Bizukojc and Ledakowicz 2010; Casas Lopez et al. 2005; Gao et al. 2014) and shown to influence the outcome of the process. The modern methods employed to engineer fungal morphology typically involve the addition of mineral microparticles, e.g. aluminium oxide or talc (Krull et al. 2013; Walisko et al. 2012, 2015). This approach is referred to as the microparticle-enhanced cultivation (MPEC) and has been successfully employed for enhancing lovastatin production (Gonciarz et al. 2016; Gonciarz and Bizukojc 2014). Even though the MPEC-based experiments were not yet tested with regard to itaconic acid production, the study of Gyamerah (1995a) involved a somewhat comparable strategy. In this work, the medium was supplemented with CaSO_4_ at the concentration beyond its solubility limit. The presence of insoluble CaSO_4_ led to the formation of small and frayed pellets. As a result, the improved production of the target metabolite was observed (Gyamerah 1995a).

## Use of glucose and lactose as carbon sources

It is generally accepted that the highest yields of itaconic acids are reached with glucose as the carbon source (Klement and Buchs 2013; Okabe et al. 2009). Glucose is rapidly metabolized via glycolysis and the resulting influx of carbon directly feeds the TCA cycle, which in turn is responsible for *cis*-aconitic and itaconic acid production. Thus, high yields of itaconic acid obtained in the presence of glucose as the carbon source can be to some extent associated with the architecture of the underlying metabolic pathways (Mondala 2015). In order to decrease the manufacturing costs, other carbon sources, e.g. beet molasses, corn starch and sago starch, were also considered as alternatives to pure glucose (Dwiarti et al. 2007; Nubel and Ratajak 1962; Petruccioli et al. 1999; Reddy and Singh 2002; Yahiro et al. 1997).

Lai et al. (2007) demonstrated that lactose, a slowly degradable carbon source, is preferred over glucose if lovastatin is the target metabolite, whereas the opposite preference was observed for itaconic acid. As already mentioned in the introduction, it is the only published report on the simultaneous biosynthesis of these two metabolites by a single strain of *A. terreus* (Lai et al. 2007). The final steps of lovastatin biosynthetic pathways are more distant from the core growth-associated primary metabolic machinery of the cell, what may be associated with the less preferable utilization of rapidly degradable glucose in this case.

Glycerol is another example of a carbon source widely used in lovastatin production (Abd Rahim et al. 2015; Jia et al. 2009; Lai et al. 2003; Manzoni et al. 1998; Pecyna and Bizukojc 2011). It was previously shown that glycerol is utilized by *A. terreus* at a lower rate than fructose, but still not as slowly as lactose (Casas Lopez et al. 2003).

## Use of ammonium salts as nitrogen sources

Ammonium salts, namely ammonium nitrate and ammonium sulfate, were used as sources of nitrogen in a majority of experimental efforts focused on itaconic acid production (*inter alia*: Gyamerah 1995a; Hevekerl et al. 2014b; Karaffa et al. 2015; Kautola et al. 1991; Kuenz et al. 2012; Nelson et al. 1952; Park et al. 1993; Riscaldati et al. 2000). Nelson et al. (1952) pointed out specifically that the consumption of ammonium ions originating from ammonium sulfate releases sulfuric acid and facilitates pH control by acidification of the broth. On the other hand, the decrease of pH should be continuously monitored in order to prevent growth inhibition due to the very high concentration of hydrogen ions.

In contrast to the common utilization of ammonium salts during itaconic acid production, their use is definitely not recommended if lovastatin is the target metabolite. Hajjaj et al. (2001) observed very poor titers of this metabolite when ammonium salts were applied as nitrogen sources. This was in agreement with the observations of Lai et al. (2003), who noted strong inhibition of lovastatin biosynthesis in the presence of ammonium sulfate. The explanation of this behavior is that the assimilation of ammonium ions is associated with the release of acids what, consequently, lowers the pH of the broth. In other words, the pH is shifted away from the values favoring lovastatin production.

## Outlook

The results of bioprocess-related studies clearly demonstrate that the conditions favoring the formation of itaconic acid and lovastatin are not identical, especially with respect to extracellular pH and the substrate preference. It is possible that their biosynthesis is inversely regulated in response to certain environmental stimuli. However, these two metabolites are markedly different in terms of chemical structure, biosynthetic origin, regulation and maximal reported titers. Itaconic acid is related to primary metabolism, whereas lovastatin is a textbook example of a secondary metabolite. Considering the metabolic and regulatory differences, the existence of common regulation for itaconic acid and lovastatin biosynthesis is rather unlikely despite the adjacency of the corresponding gene clusters. The biochemical investigation of multiple strains capable of performing simultaneous production of these two metabolites would surely provide novel insights in this regard. Still, the molecular regulatory mechanisms behind the biosynthesis of itaconic acid and lovastatin remain enigmatic and need to be addressed in future experiments. Considering the fact that the pH preference behind itaconic acid formation is different in *A. terreus* than in *U. maydis*, there is a chance these unique regulatory mechanisms were evolutionarily shaped in concert with the formation of lovastatin/itaconic acid biosynthetic genomic region.
